# Editorial: Organ preservation for rectal cancer patients

**DOI:** 10.3389/fsurg.2025.1600115

**Published:** 2025-04-25

**Authors:** Ionut Negoi, John R. T. Monson, Leonardo Bustamante-Lopez, Zoe Garoufalia, Vito D'Andrea, Sameh Hany Emile

**Affiliations:** ^1^ Department of General Surgery, Clinical Emergency Hospital of Bucharest, Carol Davila University of Medicine and Pharmacy Bucharest, Bucharest, Romania; ^2^ Department of Colorectal Surgery, Northshore University Hospital, Northwell Health, New York, NY, United States; ^3^ Department of Colon & Rectum Surgery, AdventHealth, Orlando, FL, United States; ^4^ Surgical Health Outcomes Consortium (SHOC), Advent Health Digestive Institute, Orlando, FL, United States; ^5^ Colorectal Surgery Department, Ellen Leifer Shulman and Steven Shulman Digestive Disease Center, Cleveland Clinic Florida, Weston, FL, United States; ^6^ Department of Surgery, Sapienza University of Rome, Rome, Italy

**Keywords:** rectal cancer, organ preservation, total neoadjuvant therapy, quality of life, nonoperative management

**Editorial on the Research Topic**
Organ preservation for rectal cancer patients

## Introduction

Although radical resection of rectal cancer is often curative, it can result in significant long-term functional impairment and morbidity (1). Accordingly, there has been a growing interest in organ-preserving approaches over the last decade with the aim of avoiding these morbidities without compromising oncologic outcomes (2–4). The “watch-and-wait” nonoperative management strategy, first pioneered by Habr-Gama et al., involves deferring surgery in patients who achieve a clinical complete response (cCR) after neoadjuvant therapy (5). Since the initial report in 2004 demonstrating the feasibility of watch-and-wait, multiple studies have confirmed that select patients can safely forgo surgery and still attain excellent long-term survival under strict surveillance protocols (6).

The aim of the current research is to bring additional real-world perception and evidence on the outcome of organ preservation in the treatment of rectal cancer.

## Contributing articles

In the research topic “*Organ Preservation for Rectal Cancer Patients*” six articles are included.

The current special issue highlights that organ preservation is attractive, but optimal rectal cancer management extends beyond a single approach, encompassing various prognostic factors and advanced surgical techniques. Although “watch-and-wait” strategies following clinical complete response offer functional benefits, not all patients qualify, highlighting the necessity of individualizing treatment based on anatomical and biological tumor characteristics (Dai et al.). The clinical trial by Dai et al. included 100 patients treated with total neoadjuvant therapy (TNT), and recurrence and cCR were analyzed. cCR was achieved in 25 (24%) patients. In the multivariate analysis, CRM involvement was an independent predictor of recurrence after cCR.

Tumor budding, a histological marker, significantly affects prognosis; patients with high-grade tumor budding exhibit worse overall and disease-free survival, emphasizing the critical role of pathology in clinical decision-making after neoadjuvant therapy (Rafiee et al.). A systematic review of eight studies by Rafiee et al. showed that tumor budding (TB) is a negative prognosticator of overall (3.24, 95% CI: 1.71–6.16) and disease-free (2.54, 95% CI: 1.56–4.15) survival in patients with rectal cancer treated with neoadjuvant therapy.

Accurate evaluation of complete clinical response is challenging, yet pivotal. Techniques such as transanal multipoint full-layer puncture (TMFP) biopsy are being explored to enhance the precision of cCR assessments, potentially reducing the false negatives associated with superficial biopsies (Liu et al.). Liu et al. assessed the role of transanal multipoint full-layer puncture biopsy (TMFP) to improve the accuracy of cCR and found that although challenging, this technique might be promising.

Furthermore, adjuvant chemotherapy remains beneficial for selected patients with good responses (ypT0-2N0), demonstrating improved overall survival and reduction in distant metastasis rates, underscoring the importance of systemic therapy, even after successful neoadjuvant treatment (Yang et al.).

Yang et al. reviewed 18 studies investigating the role of adjuvant chemotherapy after neoadjuvant chemoradiotherapy in ypT0-2, N0 rectal cancer and found that is has a beneficial role as it improved overall survival by 89% (OR = 1.89, 95% CI: 1.13–3.19).

Innovative local treatments, such as cryotherapy, also contribute to the multimodal toolkit, offering minimally invasive options for selected low rectal tumors, potentially preserving sphincter function without sacrificing oncologic outcomes (Jiang et al.).

Jiang et al. reviewed the role of cryotherapy in the treatment of low rectal tumors and presented their experience with this technique.

Moreover, immunotherapy combined with total neoadjuvant therapy (TNT) may dramatically enhance tumor response, enabling sphincter preservation even in traditionally challenging locally advanced cases, as demonstrated in a recent case successfully managed with robotic ultra-low anterior resection (ULAR) (Pi et al.). Pi et al. presented a case report of a 26-year-old patient with T4bN1bM0 low rectal cancer treated with TNT and immunotherapy, followed by sphincter-saving resection with robotic-assisted ultra-low rectal anterior resection with lateral lymph node dissection. The authors also reviewed the existing literature.

Despite these advances, radical surgery remains essential when organ preservation is not viable. Total mesorectal excision (TME) continues to be the cornerstone technique for curative resection, often combined with lateral lymph node dissection in selected cases with lateral nodal involvement, significantly reducing recurrence (7).

ULAR, sometimes enhanced by intersphincteric resection, facilitates sphincter-sparing resection even in ultra-low tumors, offering equivalent oncological outcomes to abdominoperineal resection (APR) but with distinct quality-of-life implications due to frequent postoperative bowel dysfunction (1, 8).

Robotic-assisted techniques increasingly support precision in pelvic surgery, enhancing the feasibility and safety of complex ULAR procedures (Pi et al.).

Thus, comprehensive multimodal management integrating accurate prognostication, tailored systemic therapies, advanced local techniques, and meticulous surgical strategies remains essential to optimize oncological and functional outcomes in patients with rectal cancer (Yang et al.).

## Discussion

Modern multidisciplinary management of rectal cancer increasingly seeks to balance oncologic cure with the preservation of anorectal function and quality of life (9). Patients with rectal cancer typically receive neoadjuvant chemoradiation, and up to 20%–30% may achieve a pathological complete response (pCR), with higher rates observed after TNT (10). Experienced teams may discuss the option for nonoperative management (NOM) in patients who achieved cCR. Approximately one-third of patients managed nonoperatively experience local tumor regrowth, but most of these recurrences can be successfully salvaged with delayed surgery, yielding comparable overall survival to immediate TME (11). Indeed, long-term outcomes such as disease-specific survival have been equivalent between carefully selected watch-and-wait patients and those undergoing standard resection after complete response (6).

The integration of immunotherapy into neoadjuvant regimens has opened new possibilities for organ preservation in rectal cancer (12). Mismatch repair-deficient (dMMR/MSI-H) rectal cancers have shown particularly dramatic responses to PD-1 checkpoint blockade. In a recent trial, all patients with locally advanced MSI-H rectal tumors achieved cCR with anti–PD-1 therapy alone, obviating the need for chemoradiation or surgery (12). Conversely, microsatellite-stable tumors respond less often to immunotherapy; however, combining standard neoadjuvant therapy with immunotherapy may increase the organ preservation rates. Larger studies are needed to validate this strategy (13).

## Conclusion

Evidence indicates that in carefully selected patients, deferring radical surgery is feasible and oncologically safe, particularly when experienced multidisciplinary multidisciplinary teams and rigorous surveillance protocols are in place. Ongoing innovations in neoadjuvant therapy, including TNT and immunotherapy, are expected to further increase the complete response rates and broaden the pool of patients eligible for NOM. At the same time, improvements in response assessment and risk stratification are critical to ensure that organ preservation is offered only when oncologically appropriate. Prospective trials and translational research will continue to refine patient selection and personalized treatment, with the goal of maximizing cure while preserving organ function, whenever possible.
